# BHLHE41 Overexpression Alleviates the Malignant Behavior of Colon Cancer Cells Induced by Hypoxia via Modulating HIF-1*α*/EMT Pathway

**DOI:** 10.1155/2022/6972331

**Published:** 2022-05-16

**Authors:** Sheng Chen, Quan-Jin Dong, Zi-Ang Wan, Shan Gao, Shi-Liang Tu, Rui Chai

**Affiliations:** General Surgery, Cancer Center, Department of Colorectal Surgery, Zhejiang Provincial People's Hospital, Affiliated People's Hospital of Hangzhou Medical College, Hangzhou City, Zhejiang Province 310014, China

## Abstract

**Objective:**

BHLHE41 has been shown to be a marker of tumorigenesis. Colon cancer (CC) is a common malignant tumor of colonic mucosa. This study mainly explored the mechanism of BHLHE41 in alleviating malignant behavior of hypoxia-induced CC cells.

**Methods:**

The levels of BHLHE41 in CC and normal cell lines were tested by Western blot and qRT-PCR. After, CC cells were subjected to hypoxia treatment and BHLHE41 overexpression transfection, and the BHLHE41 expression, the effect of BHLHE41 on CC cell viability, apoptosis, migration, and invasion and cell cycle were tested by qRT-PCR and relevant cell functional experiments. HIF-1*α* and epithelial-mesenchymal transition- (EMT-) related proteins were tested by Western blot. Moreover, CC tumor-bearing model was established in nude mice, and the effect of BHLHE41 on the tumor was evaluated by measuring the tumor volume and weight. Then, the expressions of BHLHE41 and EMT-related proteins were detected by immunohistochemistry and Western blot.

**Results:**

Western blot and qRT-PCR showed that BHLHE41 was lowly expressed in CC cells. BHLHE41 overexpression could inhibit the hypoxia-induced CC cell viability, migration, and invasion, induce apoptosis, and alter cell cycle. Besides, BHLHE41 overexpression could enhance the levels of E-cadherin but reduce the levels of HIF-1*α*, N-cadherin, vimentin, and MMP9 in hypoxia-induced CC cells. Moreover, BHLHE41 overexpression reduced tumor volume, weight, and EMT-related proteins levels in tumor tissues.

**Conclusions:**

BHLHE41 overexpression could mitigate the malignant behavior of hypoxia-induced CC via modulating the HIF-1*α*/EMT pathway.

## 1. Introduction

Colon cancer (CC) is a common malignant tumor of the digestive tract that occurs in the colon. It is often asymptomatic in the early stage and manifests as abdominal pain and blood in the stool in the middle and late stages [1]. According to statistics from the World Cancer Database in 2018, the incidence of CC has exceeded 1 million, and the mortality rate in developed countries was as high as 33% [2]. The occurrence of CC has always been considered as a common disease in middle-aged and elderly people, while currently, CC incidence is on the rise among young adults, which is related to human dietary structure, exercise, bad living habits, and other risk factors [3]. At present, the treatment of CC is mainly surgical resection supplemented by radiotherapy and chemotherapy, gene therapy, and molecular targeted therapy, while the risk of the high postoperative recurrence rate still impedes the prognosis of CC patients [4–6].

BHLHE41 is a 482 amino acid helix-loop-helix transcription factor located on human chromosome 12q12.1 [7]. BHLHE41, also known as SHARP-1, DEC2, or BHLHB3, is a member of the DEC subfamily in the bHLH gene family [8]. In our study of clinical CC and paired metastatic tissues, the gene chip technology and qRT-PCR validation results suggested that BHLHE41 was rarely expressed in CC metastatic tissues [9]. Therefore, the BHLHE41 was chosen as the target molecule in this study. The dysregulation of the transcription level of BHLHE41 has been used as a marker for the several cancers. Sato et al. found that BHLHE41 was lowly expressed in pancreatic cancer tissues, and overexpression of BHLHE41 attenuates TGF-*β*-induced migration and invasion of pancreatic cancer cells and inhibits the progression of pancreatic cancer [10].

Hypoxia of tumor tissue has been corroborated to be an important reason in leading the tumor malignant development, drug resistance, and the metastasis of recurrent lesions [11]. In the hypoxic microenvironment, tumor cells highly express hypoxia-inducible factor-1 (HIF-1*α*), which promotes the tumor cell heterogeneity and tumor angiogenesis, as well as tumor cell infiltration and metastasis [12]. Epithelial-mesenchymal transition (EMT) is involved in the initiation process of tumor malignant phenotype transformation and is a key step in tumor metastasis [13]. Studies have found that under hypoxia or normoxia, EMT can be regulated by HIF-1*α* [14]. The promoter region of BHLHE41 contains hypoxia response elements, and its expression is also regulated by HIF-1*α* [15]. In endometrial cancer, BHLHE41 mitigated the invasion of tumor cells by restraining the EMT pathway [16]. However, the role of BHLHE41 in hypoxia-induced CC has not been reported. Therefore, the role of BHLHE41 in relieving hypoxia-induced CC remains to be further investigated.

Hence, based on the construction of a hypoxia-induced CC cell model *in vitro* and a CC tumor xenografts model *in vivo*, this study was aimed at exploring that whether BHLHE41 overexpression could alleviate the malignant behavior of hypoxia-induced CC by regulating the HIF-1*α*/EMT pathway. This study hopes to provide a novel clue of BHLHE41 in CC therapy.

## 2. Materials and Methods

### 2.1. Cell Culture

Human normal colonic epithelial cell line (NCM460, C1227), human colon cancer cell lines (Lovo, C1019), and HCT116 (C1125) were acquired from WHELAB (China). NCM460 cells were grown in an epithelial cell medium (M1005A, WHELAB, China). Lovo and HCT116 cells were cultured in F-12 K complete medium (M0511A, WHELAB, China). Then, the above cells were cultured in incubator (BB150, ThermoFisher, USA). For hypoxia treatment, the CC cells were cultured for 9 h at 37°C in a hypoxic chamber with 1% O_2_, 5% CO_2_, and 94% N_2_ [17].

### 2.2. Cell Transfection

BHLHE41 overexpression was constructed by a pcDNA3.1 (+) vector (VT1001, YouBio, China), and the empty vector was taken as negative control (NC). BHLHE41 overexpression and NC were transfected into hypoxia-treated CC cells (1 × 10^5^) by Lipofectamine™ 2000 (11668030, ThermoFisher Scientific, USA).

### 2.3. Western Blot

Tumor tissues, NCM460, and CC cells were collected and lysed by a RIPA buffer (G2002, Servicebio, China). Then, the total protein concentration was determined by a BCA kit (G2026, Servicebio, China). Protein samples were separated by electrophoresis on 10% SDS-PAGE gels before semidry transfer to PVDF membrane (10600023, GE Healthcare Life, USA) and then sealed with 5% BSA. After reaction with primary antibodies overnight at 4°C, they were placed in the incubation box containing secondary antibody at 37°C for 1 h. Lastly, we utilized the color reagent (E266188, Aladdin, China) to visualize the bound antibodies which were exposed in a gel imaging system (610020-9Q, Clinx, China). The primary antibodies of anti-BHLHE41 antibody (AF0442), anti-HIF-1*α* antibody (AF1009), anti-E-cadherin antibody (AF0131), anti-N-cadherin antibody (AF4039), anti-vimentin antibody (AF7013), anti-MMP9 antibody (AF5228), and anti-GAPDH antibody (AF7021) were obtained from the Affinity (USA).

### 2.4. QRT-PCR

Total RNA was extracted from cells by the Trizol reagent (abs60031, absin, China), then it was reversely transcribed to cDNA by the cDNA synthesis kit (11483188001, Roche, USA). Then, QRT-PCR was performed by the SYBR Green PCR Mastermix (2×, SR1110, Solarbio, China) in a PCR system (QuantStudio 5, ABI, USA). GAPDH was served as the normalization control. The results were calculated by the 2^-*ΔΔ*Ct^ method. The primers were as follows: BHLHE41 forward, 5′-AAGGAGCATGAAACGAGACGA-3′ and reverse, 5′-CTCGGTTAAGGCGGTTAAAGC-3′; GAPDH forward, 5′-TGTGGGCATCAATGGATTTGG-3′ and reverse, 5′-ACACCATGTATTCCGGGTCAAT-3′.

### 2.5. Cell Viability Assay

The CCK-8 kit (HY-K0301, MCE, USA) was used to assess the CC cell viability. The treated CC cells were cultured for 24 h, 48 h, 72 h, and 96 h, respectively. Then, the cells were treated with 10 *μ*L CCK-8 solution for 3 h. Finally, a microplate reader (CMaxPlus, MD, USA) was used to estimate the absorbance (450 nm).

### 2.6. Flow Cytometry Assay

Annexin V-FITC/PI kit (556547, BD Biosciences, USA) was used to detect the apoptosis of CC cells. Cells were collected then washed twice with PBS and centrifuged to dilute cells with buffer. Each sample tube was reacted with 5 *μ*L Annexin V-FITC and 10 *μ*L PI at 37°C for 15 min in a dark room. Finally, 400 *μ*L of buffer was added and the apoptosis rate of CC cells was detected by flow cytometry (LSRII, BD Biosciences, USA). Then, the cell suspensions of the HCT116 and Lovo cells in the logarithmic phase were inoculated into plates, and the culture plates were precultured for 24 h. The cell cycle was detected by flow cytometry after 24 h.

### 2.7. Cell Migration and Invasion Assay

When the cell migration experiment was performed, a Transwell chamber (3422, Corning, USA) was put into a 24-well culture plate. For invasion experiment, the diluted matrigel (356234, BD Biosciences, USA) was coated in the Transwell chamber overnight at 4°C. For migration (invasion) experiment, the Transwell chamber was injected into 200 *μ*L cell suspension without FBS and the lower chamber was filled with 600 *μ*L medium containing 10% FBS. After 24 h, we utilized cotton swabs to wipe off the cells inside the upper chamber. The cells on the lower membrane surface were fixed with methanol for 10 min, which were then stained with 0.1% crystal violet dye solution (C0121, Beyotime, China) for 30 min. Finally, photographed and counted the number of cells migrated and invaded.

### 2.8. Nude Mice Tumorigenicity Assay

12 SPF male BALB/c nude mice (4-6 weeks old and 14-18 g) were acquired from Shanghai SLAC Laboratory Animal Co., Ltd. (Certificate No. SCXK (Hu)2017-0005, Shanghai, China). All nude mice were reared in the SPF-level feeding center of Hangzhou Eyong Biotechnological Co., Ltd. Animal Experiment Center (Certificate No. SYXK (Zhe)2020-0024) and housed in an environment with temperature of 25 ± 2°C and the humidity of 50 ± 5%.

All nude mice were randomly divided into 2 groups (*n* = 6): NC and BHLHE41 groups. HCT116 cells (5 × 10^6^) transfected with NC or BHLHE41 overexpression were injected subcutaneously into the right armpit of the mice. The tumor volume was gauged every 3 days until the 21^th^ day. After 21^th^ day, the nude mice were euthanized by cervical dislocation with intraperitoneal anesthesia of 1% pentobarbital sodium (100 *μ*L, P-010, Supelco, USA) [18]. Finally, the tumor was removed and weighed. The tumor tissues were stored for follow-up experiment.

### 2.9. Immunohistochemical (IHC) assay

The fixed tumor tissues were dehydrated, embedded, and sectioned (4 *μ*m) then dewaxed, hydrated, and repaired by 0.01 M citrate buffer solution (pH 6.0, G1202, Servicebio, China). When it was reacted with 3% hydrogen peroxide for 10 min, the sections were sealed and then reacted with anti-BHLHE41 antibody overnight at 4°C. The following day, the sections were subjected to HRP-labeled secondary antibody (1 : 200, S0001, Affinity, USA) at 37°C for 1 h. Sections were developed with DAB kit (G1211, Servicebio, China), counterstained, differentiated, and returned to blue. After the sections were dehydrated and sealed, they were observed under the optical microscope.

### 2.10. Statistical Analysis

SPSS software (16.0, IBM, USA) was used for data analysis. Student *t* test was used for two group comparison, and one-way ANOVA followed by Tukey test was utilized for multiple group comparison if it was normally distributed. Kruskal-Wallis H test was utilized if it was not normally distributed. All data were described as mean ± standard deviation. *P* < 0.05 suggested that the difference was statistically significant.

## 3. Results

### 3.1. BHLHE41 Was Low Expressed in HCT116 and Lovo Cells

**Figure 1:**
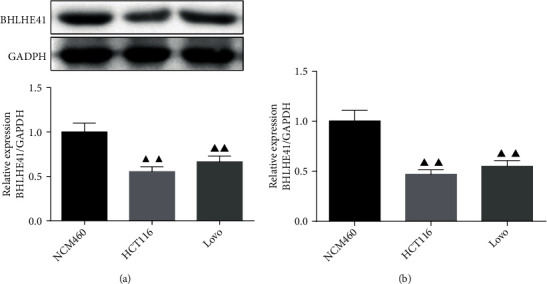
BHLHE41 was low expressed in HCT116 and Lovo cells. (a) and (b) The level of BHLHE41 in colon cancer (CC) cells and normal cells was tested by Western blot and qRT-PCR. All experiments have been performed in triplicate, and data were expressed as mean ± standard deviation (SD). ^▲▲^*P* < 0.01 vs. NCM460.

**Figure 2:**
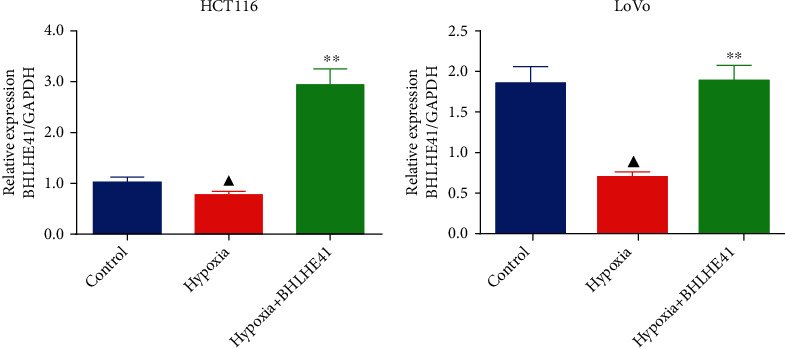
Expression of BHLHE41 mRNA in HCT116 and Lovo cells. All experiments have been performed in triplicate, and data were expressed as mean ± SD. ^∗∗^*P* < 0.01 vs. hypoxia; ^▲^*P* < 0.5, ^▲▲^*P* < 0.01 vs. control.

The expression of BHLHE41 in CC cells (HCT116 and Lovo) and normal colon epithelial cells (NCM460) was investigated by Western blot and QRT-PCR assay. The results showed that the protein and mRNA expression of BHLHE41 was decreased in CC cells (HCT116 and Lovo) relative to the NCM460 cells (Figures 1(a) and 1(b), *P* < 0.01). In addition, after BHLHE41 overexpression and NC were transfected into hypoxia-treated CC cells (HCT116 and Lovo), the result showed that the mRNA expression of the BHLHE41 was significantly elevated in hypoxia+BHLHE41 overexpression group compared to the hypoxia group of CC cells (Figure 2, *P* < 0.01).

### 3.2. BHLHE41 Overexpression Repressed the Cell Viability, Migration, and Invasion, Blocked Cell Cycle, and Induced Apoptosis in Hypoxia-Induced CC Cells

**Figure 3:**
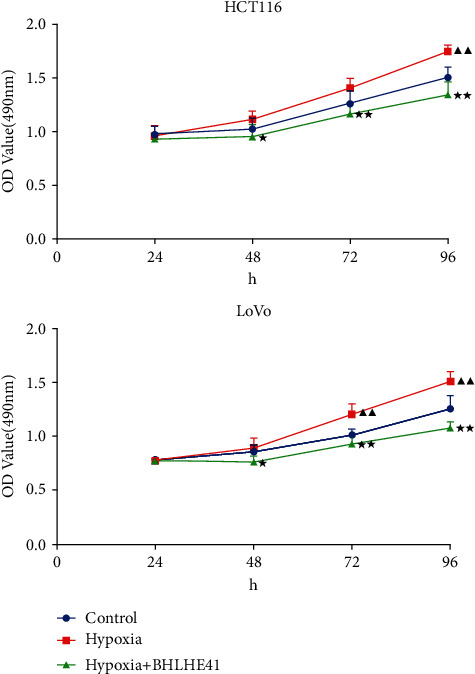
BHLHE41 overexpression repressed the cell viability in hypoxia-induced CC cells. The cell viability in hypoxia-induced CC cells was tested by CCK-8. All experiments have been performed in triplicate, and data were expressed as mean ± SD. ^∗^*P* < 0.05, ^∗∗^*P* < 0.01 vs. hypoxia; ^▲▲^*P* < 0.01 vs. control.

**Figure 4:**
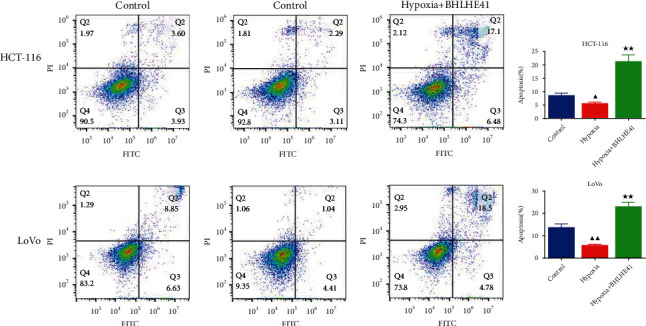
BHLHE41 overexpression induced apoptosis in hypoxia-induced CC cells. Apoptosis in hypoxia-induced CC cells was tested by flow cytometry. All experiments have been performed in triplicate, and data were expressed as mean ± SD. ^∗∗^*P* < 0.01 vs. hypoxia; ^▲▲^*P* < 0.01 vs. control.

**Figure 5:**
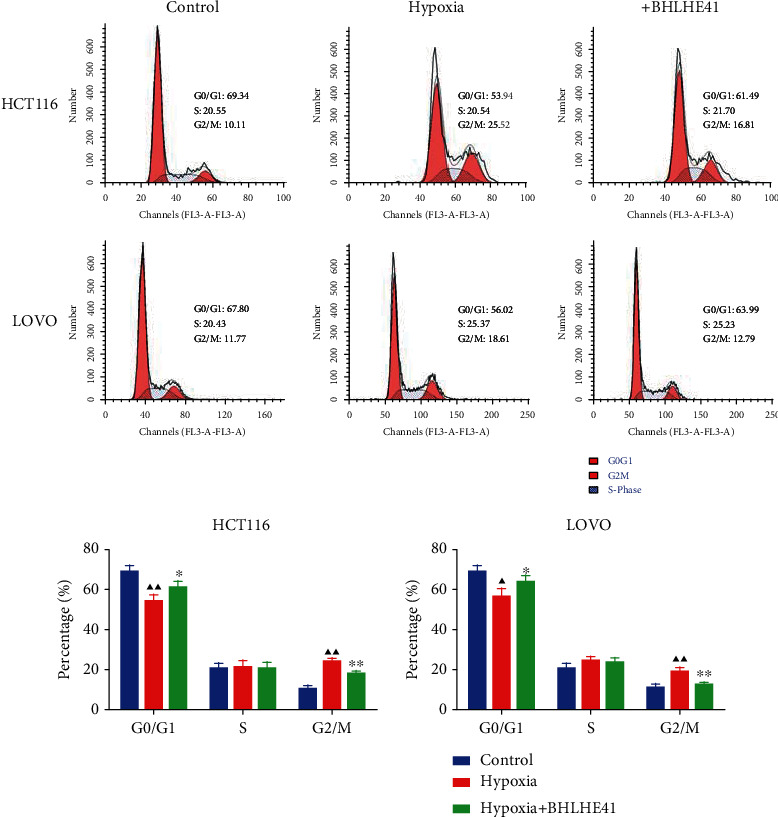
BHLHE41 overexpression blocked the HCT116 and Lovo cell cycle, and decreased the number of cells in G2/M phase. The cell cycle in hypoxia-induced CC cells was tested by flow cytometry. All experiments have been performed in triplicate and data were expressed as mean ± SD. ^∗∗^*P* < 0.01 vs. hypoxia; ^▲▲^*P* <0.01 vs. control.

**Figure 6:**
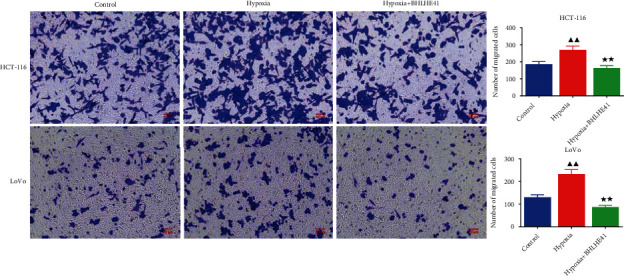
BHLHE41 overexpression repressed the migration in hypoxia-induced CC cells. The migration in hypoxia-induced CC cells was tested by Transwell assay. All experiments have been performed in triplicate, and data were expressed as mean ± SD. ^∗∗^*P* < 0.01 vs. hypoxia; ^▲▲^*P* < 0.01 vs. control.

**Figure 7:**
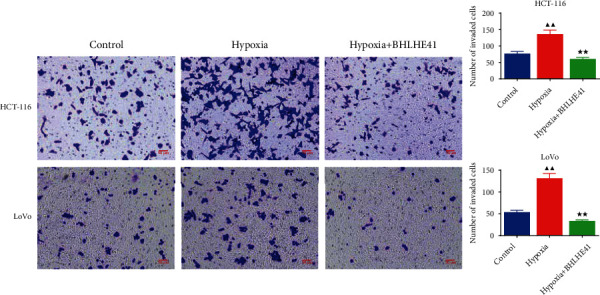
BHLHE41 overexpression repressed the invasion in hypoxia-induced CC cells. The invasion in hypoxia-induced CC cells was tested by Transwell assay. All experiments have been performed in triplicate, and data were expressed as mean ± SD. ^∗∗^*P* < 0.01 vs. hypoxia; ^▲▲^*P* < 0.01 vs. control.

To explore the role of BHLHE41 in hypoxia-induced CC cells, overexpressed BHLHE41 was transfected into CC cells, and a series of cell function experiments were performed. Hypoxia-induced CC cells (HCT116 and Lovo) increased cell viability compared with the control group, while BHLHE41 overexpression inhibited viability (Figure 3, *P* < 0.05). The results of apoptosis experiments illuminated that hypoxia repressed CC cell apoptosis, while BHLHE41 overexpression promoted hypoxia-induced CC cell apoptosis (Figure 4, *P* < 0.01). Cell cycle experiments showed that the number of cells in G0/G1 phase was significantly reduced, and the number of cells in G2/M phase was increased in hypoxia group, while BHLHE41 overexpression reversed the number of cells in G0/G1 and G2/M phases (Figure 5, *P* < 0.01). Furthermore, cell migration and invasion experiments found that hypoxia enhanced the migration and invasion ability of CC cells, whereas BHLHE41 overexpression reversed the cell migration and invasion ability (Figures 6 and 7, *P* < 0.01).

### 3.3. BHLHE41 Overexpression Weakened the Levels of the HIF-1*α* and EMT-Related Factors in Hypoxia-Induced CC Cells

**Figure 8:**
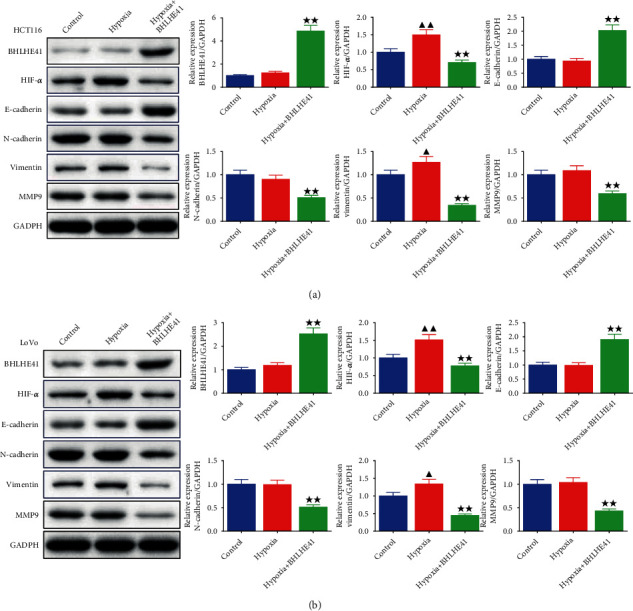
BHLHE41 overexpression weakened the HIF-1*α* and EMT-related factor levels in hypoxia-induced CC cells. (a) and (b). The BHLHE41, hypoxia-inducible factor-1alpha (HIF-1*α*), and epithelial-mesenchymal transition- (EMT-) related factor levels in hypoxia-induced CC cells were tested by Western blot. All experiments have been performed in triplicate, and data were expressed as mean ± SD. ^∗∗^*P* < 0.01 vs. hypoxia; ^▲^*P* < 0.5, ^▲▲^*P* < 0.01 vs. control.

Western blot was used to detect HIF-1*α* and EMT-related protein levels. The study found that hypoxia increased the levels of HIF-1*α* and vimentin (Figures 8(a) and 8(b), *P* < 0.05) but had no significant change in the levels of BHLHE41, E-cadherin, N-cadherin, and MMP9. Moreover, BHLHE41 overexpression significantly enhanced the levels of BHLHE41 and E-cadherin but decreased the levels of HIF-1*α*, N-cadherin, vimentin, and MMP9 in hypoxia-induced CC cells (Figures 8(a) and 8(b), *P* < 0.01).

### 3.4. BHLHE41 Overexpression Inhibited the Tumor Growth and Enhanced the Positive Expression of BHLHE41 in Tumor Tissues

**Figure 9:**
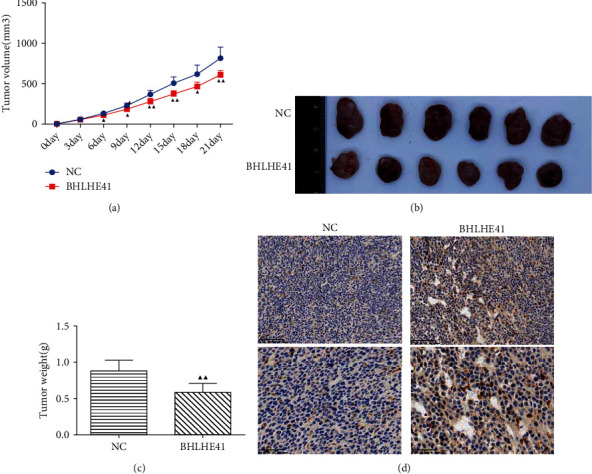
BHLHE41 overexpression attenuated tumor volume and weight and enhanced the positive expression of BHLHE41 in tumor tissues. (a) Tumor volume growth curve. (b) Tumor pictures of nude mice. (c) Tumor weight. (d) The positive expression of BHLHE41 in tumor tissues was tested by immunohistochemistry. All experiments have been performed in triplicate, and data were expressed as mean ± SD. ^▲^*P* < 0.5, ^▲▲^*P* < 0.01 vs. negative control (NC).

In order to verify the effect of BHLHE41 in CC tumors, a CC nude mouse model was established. HCT116 cells transfected to NC or BHLHE41 overexpression were injected into mice. The results discovered that BHLHE41 overexpression significantly inhibited the growth of tumor volume and tumor weight (Figures 9(a)–9(c), *P* < 0.05). In addition, the IHC assay demonstrated that BHLHE41 was also overexpressed in tumor tissues (Figure 9(d)).

### 3.5. BHLHE41 Overexpression Reduced the Level of the EMT-Related Protein in Tumor Tissues

**Figure 10:**
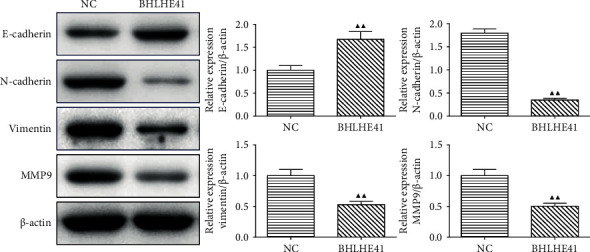
BHLHE41 overexpression alleviated the EMT-related factors level in tumor tissues. The EMT-related markers in tumor tissues were tested by Western blot. All experiments have been performed in triplicate, and data were expressed as mean ± SD. ^▲▲^*P* < 0.01 vs. NC.

The level of EMT-related proteins in tumor tissue was detected by Western blot. The results demonstrated that the overexpression of BHLHE41 significantly decreased the levels of N-cadherin, vimentin, and MMP9 and significantly increased the level of E-cadherin in tumor tissue (Figure 10, *P* < 0.01).

## 4. Discussion

Due to the rapid growth of tumors, the abnormal blood vessels inside the tumor cannot meet the oxygen demand for tumor growth, so 50% to 60% of solid tumors have hypoxic areas [19]. Studies have shown that tumor cells can continue to survive in the hypoxic microenvironment and can escape the damage caused by hypoxia, so the hypoxic environment plays a key role in the occurrence and malignant progression of tumors [20–22]. In this research, it was found that hypoxia stimulation led to the increase of cell viability, migration, and invasion and the decrease of apoptosis in CC cells. However, BHLHE41 overexpression could attenuate hypoxia-induced malignant behavior of CC cells by modulating the HIF-1*α*/EMT pathway.

The BHLHE41 gene is located on human chromosome 12q12.1, which plays a critical role in modulating cell differentiation, apoptosis, hypoxia, and immune response [23–25]. Nagata et al. found that BHLHE41 expression was lower in lung adenocarcinoma tissue than in normal lung tissue [26]. This is consistent with the findings of this study that BHLHE41 is low expressed in HCT116 and Lovo cells. In the study, it was found that the BHLHE41 mRNA expression in the hypoxia+BHLHE41 group was significantly higher than that in the hypoxia group. Furthermore, the cell function experiments confirmed that BHLHE41 overexpression inhibited the cell viability, migration, and invasion in hypoxia-induced CC cells and promoted CC cell apoptosis. The result of Zhang et al. exhibited that BHLHE41 silencing facilitated migration and invasion in breast cancer cells through activating MAPK/JNK pathway [24]. However, the result of Shen et al. found that BHLHE41 has no effect on apoptosis [9], which is inconsistent with the results of this study.

A study proved that BHLHE41 was a key regulator of invasive and metastatic phenotypes in triple-negative breast cancer, and it can bind to HIF to promote the HIF protease degradation [27]. In thyroid cancer cell lines, overexpression of BHLHE41 downregulated HIF-1*α* protein and mRNA levels and obviously repressed cell migration and invasion, revealing that BHLHE41 may play a tumor suppressor effect in thyroid cancer by modulating the HIF-1*α* pathway [28]. Hypoxia activates the HIF-1*α* signaling pathway, resulting in the activation of hundreds of downstream genes [29]. These activated genes enable tumor cells to survive in a hypoxic environment and promote invasion and metastasis of tumor cells by directly activating EMT-related genes [30]. It has been reported that EMT plays a crucial role in CC cells [31]. Moreover, the downregulation of E-cadherin and the upregulation of N-cadherin and vimentin are considered to be important factors for tumor cells to obtain EMT pathway [32, 33]. In our study, it was discovered that BHLHE41 overexpression largely enhanced the levels of BHLHE41 and E-cadherin but repressed the levels of HIF-1*α*, N-cadherin, vimentin, and MMP9 in hypoxia-induced CC cells, which was consistent with the report of BHLHE41 in breast cancer [27]. In endometrial cancer, BHLHE41 expression is downregulated, and overexpressed BHLHE41 reduces HIF-1 levels thereby attenuating endometrial cancer angiogenesis [34].

In this study, CC tumor xenografts in nude mice were performed to explore the effect of BHLHE41 overexpression in reducing hypoxia-induced malignant behavior of CC cells. It was found that BHLHE41 overexpression significantly reduced tumor volume and tumor weight and promoted the positive expression of BHLHE41 in tumor tissues. This is similar to previous results that BHLHE41 overexpression inhibits tumor growth and metastasis, as well as being positive for the EMT regulator E-cadherin [35]. In addition, BHLHE41 overexpression altered the levels of EMT-related proteins in tumor tissues, and BHLHE41 overexpression significantly decreased the levels of N-cadherin, vimentin, and MMP9 and significantly increased the levels of E-cadherin in tumor tissues, which was consistent with the results of *in vitro* experiments and Montagner et al. [27] .

In summary, this study demonstrated that overexpression of BHLHE41 could inhibit the activity, migration, and invasion and promote apoptosis of CC cells by regulating the HIF-1*α*/EMT pathway. In addition, it was found that in animal experiments, overexpression of BHLHE41 can also reduce the volume and mass of tumor mice. It provides a new idea for BHLHE41 in alleviating hypoxia-induced CC malignant behavior. However, this study also has some limitations. The expression rate of BHLHE41 in human colon cancer in clinical staining test requires further study.

## Data Availability

All data generated or analyzed during this study are included in this article.
